# SARS-CoV-2 and the Use of Chloroquine as an Antiviral Treatment

**DOI:** 10.3389/fmed.2020.00184

**Published:** 2020-04-24

**Authors:** Mathieu E. Rebeaud, Florian Zores

**Affiliations:** ^1^DBMV, Faculty of Biology and Medicine, University of Lausanne, Lausanne, Switzerland; ^2^Groupe médical spécialisé, Strasbourg, France

**Keywords:** COVID-19, SARS-CoV-2, chloroquine, antiviral, 2019-nCoV

## Introduction

In December 2019, a newly discovered SARS-CoV-2 virus emerged from China and propagated worldwide as a pandemic, becoming a major global public health issue. Different publications have discussed the possible efficacy of the antimalarial drug chloroquine (CQ) and its derivatives as a possible treatment against the disease, and, as the drug has often been recommended, we would like to shed a light on the previous experiments and trials conducted with CQ and its derivatives on several viruses, the outcomes being based on *in vitro* and *in vivo* results, and call for a well-designed clinical evaluation.

## Chloroquine, Hydroxychloroquine, and Other Quinine-Derivative Drugs

As a semisynthetic derivative of quinine, CQ has for decades been the drug of choice to treat malaria because of its relative safety, good efficacy, and for being relatively inexpensive. CQ is a lysosome-penetrating antimalarial drug that neutralizes lysosomal acidification and prevents autophagosomal degradation. Hydroxychloroquine (HCQ) is a 4-aminoquinoline that differs by the addition of a hydroxyl group, decreasing its toxicity while conserving its efficacy. Nevertheless, CQ has a narrow therapeutic window and can cause life-threating cardiovascular issues, documented since the early 80s, especially for patients with underlying cardiac diseases (1). Cardiomyopathies, fatal arrhythmia, or even complete heart block have been described for 40 years, for chronic as well as acute treatment, even in patients with normal underlying cardiac function (2, 3). Another issue is represented by the possibility of vision-threatening toxic retinopathy (4). Thus, major contraindications are related to ocular (pre-existing maculopathy and retinopathy) and cardiac abnormalities [recent myocardial infarction and heart failure, corrected QT interval (QTc) >500 ms] but also include hypersensitivity to the active ingredient, porphyria, or glucose-6-phosphate dehydrogenase (G6PD) deficiency. It is also not recommended to combine these drugs with macrolides such as Azithromycin, which are known to have a synergistic effect on QTc prolongation, as QTc prolongation is associated with an increased risk of life-threatening arrhythmia (5). For the same reason, CQ and HCQ should not be used concomitantly with lopinavir/ritonavir and remdesivir. However, these drugs are not contraindicated during pregnancy (6).

## SARS-CoV-2

In December 2019, COVID19, a novel pneumonia caused by a previously unknown pathogen, emerged in Wuhan, China. The pathogen was soon identified as a novel coronavirus (2019-nCoV, later called SARS-CoV-2), closely related to the one responsible for severe acute respiratory syndrome SARS (SARS-CoV). SARS-CoV-2 infection is triggered by the binding of the spike protein of the virus to angiotensin converting enzyme 2 (ACE2), which is highly expressed in the heart, gut, oral cavity, and lungs (7–9). SARS-CoV-2 mainly invades alveolar epithelial cells, resulting in respiratory symptoms. Briefly, in the cases where it is required, the median duration of hospitalization is 12 days (mean, 12.8) (10). Whereas, many people infected by SARS-CoV-2 develop mild, inconsequential respiratory symptoms, some individuals may develop more severe forms. During hospital stay, pneumonia is the most frequent diagnosis (91.1%), followed by acute respiratory distress syndrome (ARDS) (3.4%), but other organ dysfunctions can occur, leading to shock, multiple organ failure, and eventually death. Despite a lower case fatality rate than either SARS-CoV or Middle East respiratory syndrome-related coronavirus (MERS-CoV) (11, 12), the high number of infected patients can lead to a critical healthcare crisis, as depicted recently in China, Italy, France, and other countries. Currently, there is no specific treatment against the new virus other than supportive care. Therefore, identifying effective agents is urgently needed, either to combat the acute and severe forms of the disease, or to reduce infectiousness in less severe forms in order to reduce the burden for healthcare systems.

## Chloroquine as a COVID-19 Treatment: *in vitro* and *in vivo* Data

CQ efficacy has been tested *in vitro* since the late 60s in different animal cells and viruses (13, 14). Thirty years ago, when comparing *in vitro* and *in vivo* trials and experiments, Hellgren et al. (15) already raised doubts concerning extrapolation drawn between the two systems and bench to bedside reproducibility. The sensitivity and therapeutic range of CQ, even in antimalarial treatment, cannot be easily derived from *in vitro* to *in vivo*. Hellgren et al. studied the *in vivo* response to a standard (25 mg/kg) dosage of chloroquine in a group of semi-immunized children from Tanzania. The EC99 (99% inhibition of schizont maturation) *in vitro* was 2.7 μg/L, and *in vivo* minimum inhibitory concentrations (MIC) median values were 44.29 (13–202; *n* = 22) μg/l, for a clearance of parasites, but recrudescence 1–4 weeks later and 237 (range 133–261; *n* = 7) μg/L for a response when parasitemia failed to clear after 1 week of treatment.

CQ, by inhibiting pH-dependent steps of the replication of several viruses, has already been quite extensively tested *in vitro* and *in vivo* on different virus strains: African swine fever virus (16), HIV (17), SARS-CoV (18, 19), Influenza A (20), Chikungunya (21), Ebola (22, 23), Zika (24), and, recently, on SARS-CoV-2 (25–27). Treatment with CQ has showed interesting results but also strong differences of application between live animals and cell lines. The major conclusion of these studies was that, if CQ exhibited promising results on virus and cells, the *in vivo* application is not that straightforward. In the case of Influenza A, the effectiveness of CQ *in vitro* on limiting the replication of viruses does not extend to *in vivo* models of influenza. For Ebola virus, the replication was inhibited by chloroquine *in vitro* but failed to protect Guinea pigs, mice, and hamsters. The most important warning on the difficulties to translate *in vitro* success into clinical reality is provided by the paradoxical results against Chikungunya. Despite inhibiting Chikungunya *in vitro*, CQ decreases cytokines levels and thus delays adaptive immune responses (28). De Lamballerie et al. (21) subsequently showed in a double blind randomized control trial that CQ has no more effect than a placebo in the acute phase but, in spite of this, increases late onset symptoms.

## Discussion

Despite these discrepancies between *in vitro* and *in vivo* data on all other tested viruses, CQ has been called a potential effective treatment for COVID-19. Many commentators have urged the use of CQ to lower the COVID-19 mortality rate after the publication of a Chinese expert consensus on CQ use in COVID-19 (29) and the result of a first trial (30). Nevertheless, this consensus did not provide any clinical data and is only based on *in vitro* assumptions. The trial by Gautret et al. suffers from several strong methodological problems, which preclude any conclusion (31). To date, only one small randomized unblinded prospective trial of 30 patients comparing CQ + standard of care vs. standard of care alone has been published and failed to show a difference between both arms for the primary endpoint [negative conversion rate of COVID-19 nucleic acid in respiratory pharyngeal swab on day 7 after randomization (32)].

Like in other major previous viral outbreak, treatment of COVID-19 is largely based on off-label, and compassionate therapies based on physiopathological or *in vitro* considerations. Likewise, is CQ, as suggested, a good treatment option given that it is presented as a well-known drug that has been used for decades? Thus, it is assumed it cannot be worse than the disease itself. For ethical reasons, this statement can equally be used to refute the need of a trial or the need of a control arm. Nevertheless, at the end of March, we counted 30 (Table 1) ongoing trials listed in Chinese, European, and US clinical trial registries, with a large variety found in the design or endpoint (EP).

**Table 1:** List of clinical trials listed in Chinese, European, and US clinical trial registries.

	**Nb. of studies**	**Nb. of patients (median [IQR25-75])**	Control arm^*^			**Endpoint**					
			**SOC**	**Placebo**	**Active treatment**	**None**	**Viral surrogate EP**	**Length of stay**	**Clinical status**	**Time to improvement**	**Mortality**
Cohort study	7 (23,3%)	100.0 [22.5; 129.0]	3	0	2 (antiviral)	2	4	2	1	0	0
Open-label randomized trial	17 (56,7%)	150.0 [100.0; 647.5]	10	0	10 (antiviral, immunomodulator, hydroxyCQ vs. CQ)	0	5	0	4	6	3
Single blind randomized trial	2 (6,7%)	125.0 [112.5; 137;5]	1	1	0	0	1	1	0	0	0
Double blind randomized trial	4 (13,3%)	278.0 [224;5; 372;5]	0	4	0	0	2	0	0	0	2
Total	30	150.0 [100.0; 440.0]	14	5	12	2	12	3	5	6	5

* *some trials can have multiple control arms*.

Several drugs have failed in the past to confirm, in a randomized control trial, a putative efficacy seen in observational or phase 2 studies. Some have even been found to increase mortality despite promising results on physiological endpoints and safe use in other diseases. Since CQ has well-known potentially life-threatening cardiac side effects due to its quinidinic-like properties and the cardiac involvement of COVID-19 is now well-documented (33), the CAST study example (34) is of particular interest. It underlines the deleterious effect of class 1 antiarrhythmics in case of cardiac ischemia or left ventricular dysfunction despite its apparent safety in other medical conditions.

Some argue the mortality rate is too high to ethically run a controlled trial. Firstly, this assumes placebo is always worse than active treatment (that is untrue). Secondly, even if the global mortality rate is perceived as high because of the large number of infected patients, it is far lower than the terrible outcome associated with out-of-hospital cardiac arrest. Nevertheless, a randomized double-blind trial was performed to establish epinephrine effectiveness in out-of-hospital arrest (35). As reminded by Kalil in a recent paper (36), randomized control trials are the only way to precisely determine the harms of the drug and its safety in all medical situation and in the precise context of COVID-19. Only a quarter of ongoing trials are cohort studies, and a vast majority are controlled ones and will probably provide a good enough level of evidence for the effectiveness and the safety of CQ in COVID-19, if EPs are well-chosen.

A valuable EP is of particular importance to establish the efficiency. The first two published trials (30, 32) used a surrogate endpoint (the viral clearance). The sensitivity of SARS-COV2 PCR is quite low (37) and it can preclude any translation of the effectiveness on viral clearance to mortality or morbidity benefits. It is easier and less expensive to show that a treatment improves a surrogate endpoint than a clinical one (like clinical status or, at best, mortality). Nevertheless, a CAST trial showed us an improvement in a surrogate endpoint does not necessary translate into a decrease in clinical events or in mortality. As demonstrated many years ago by Prasad and Cifu (38), such surrogate endpoints, especially for unblinded trials, are the way to medical reversal and can lead to patient harm. The weaker the endpoint, the stronger the trial design to avoid inconclusive results. More importantly, falsely reassuring results based on surrogate endpoints can slow down the research of an effective treatment. Concerns have been raised about enrolment in the major European randomized trial DISCOVERY because of the mediatized claimed CQ effectiveness. Nine (69.2%) of the actual ongoing studies using a viral surrogate EP are of poor methodological quality (cohort studies or open-label trials) and will hardly give a valuable answer for the therapeutic value of CQ. All-cause mortality is the ideal endpoint but can be hard to reach due to economic, temporal, and demographic considerations. EPs, such as vital status evolution or length of stay, are more pragmatic to have a rapid and quite robust answer in a randomized trial and are used by near half of ongoing studies. Nevertheless, these EPs are potentially more subjective and more subject to bias than an objective one like death (39). Thus, particular attention should be paid to the design of these trials and the definitions of theses EP when interpreting the future results.

## Conclusion

Since the late 60s, the option to use CQ and quinine derivative drugs as antivirals has been considered in a wide range of diseases (40). Based on the recent announcements of Gao et al. (25), Wang et al. (26), and Colson et al. (27), Chloroquine may be the first successful attempt to use this drug as an *in vivo* (human) antiviral.

However, despite the increased knowledge accumulated in recent decades, CQ has never been selected as a definitive or effective treatment in humans, as it failed to translate *in vitro* efficacity to *in vivo* efficiency. Moreover, the narrow therapeutic windows, along with possible side effects, have often interceded against its use. The ongoing SARS-CoV2 pandemic is a huge challenge for the whole world. Its relatively moderate mortality rate is aggravated by its high infectivity and the burden it causes on healthcare system in many countries. The will to give patients a treatment option even if proof is lacking is a human natural behavior in this time of need. Though scientific precision may seem insensitive, it is the best way to avoid harming patients. Medical history is made of unmet hopes, and potential beneficial drugs have shown at best no effectiveness and have even been associated with increased adverse events. Failure to translate *in vivo* the *in vitro* success of CQ on Chikungunya is another reminder of the need of a careful clinical evaluation. To date, no published data support the use of CQ in COVID19. Well-designed clinical trials (randomized and controlled) with valuable and less as possible subjective EPs are urgently needed to clearly establish safety and effectiveness of quinine derivatives like Chloroquine as antiviral treatments.

## Author Contributions

MR and FZ wrote the manuscript and collected the data of the existing clinical trials. All authors reviewed and approved the final version of the manuscript.

## Conflict of Interest

The authors declare that the research was conducted in the absence of any commercial or financial relationships that could be construed as a potential conflict of interest.
